# An Enantioselective Potentiometric Sensor for 2-Amino-1-Butanol Based on Chiral Porous Organic Cage CC3-R

**DOI:** 10.3390/molecules24030420

**Published:** 2019-01-24

**Authors:** Bang-Jin Wang, Ai-Hong Duan, Jun-Hui Zhang, Sheng-Ming Xie, Qiu-E Cao, Li-Ming Yuan

**Affiliations:** 1 Key Laboratory of Medicinal Chemistry for Natural Resource, Ministry of Education, School of Chemical Science and Technology, Yunnan University, Kunming 650091, China; wangbangjin711@163.com; 2 Department of Chemistry, Yunnan Normal University, Kunming 650500, China; duanaihong215@aliyun.com (A.-H.D.); zjh19861202@126.com (J.-H.Z.); xieshengming_2006@163.com (S.-M.X.)

**Keywords:** enantioselective potentiometric sensor, 2-amino-1-butanol, chiral porous organic cage, CC3-R, PVC membrane electrode

## Abstract

Porous organic cages (POCs) have attracted extensive attention due to their unique structures and tremendous application potential in numerous areas. In this study, an enantioselective potentiometric sensor composed of a polyvinyl chloride (PVC) membrane electrode modified with CC3-R POC material was used for the recognition of enantiomers of 2-amino-1-butanol. After optimisation, the developed sensor exhibited enantioselectivity toward *S*-2-amino-1-butanol ($\log K_{S,R}^{Pot}$ = −0.98) with acceptable sensitivity, and a near-Nernstian response of 25.8 ± 0.3 mV/decade within a pH range of 6.0–9.0.

## 1. Introduction

Chirality is a general phenomenon and an important characteristic in naturally occurring molecules. For instance, most amino acids are levorotatory and sugars are dextrorotatory in biological systems. Consequently, chiral discrimination has attracted tremendous attention on account of its significance in pharmaceutical, biomedicine and chemical fields. Currently, chiral discrimination can be precisely achieved in many ways including gas chromatography (GC), high-performance liquid chromatography (HPLC) and high-performance capillary electrophoresis (HPCE). Although these methods have different advantages in terms of sensitivity or applicability, they suffer similar drawbacks including complicated operation and the need for expensive equipment. By contrast, ion-selective electrodes are simple, rapid and affordable, and have been widely applied to the enantioselective recognition and detection of chiral compounds in recent years [1,2,3,4,5,6,7,8,9].

**Figure 1:**
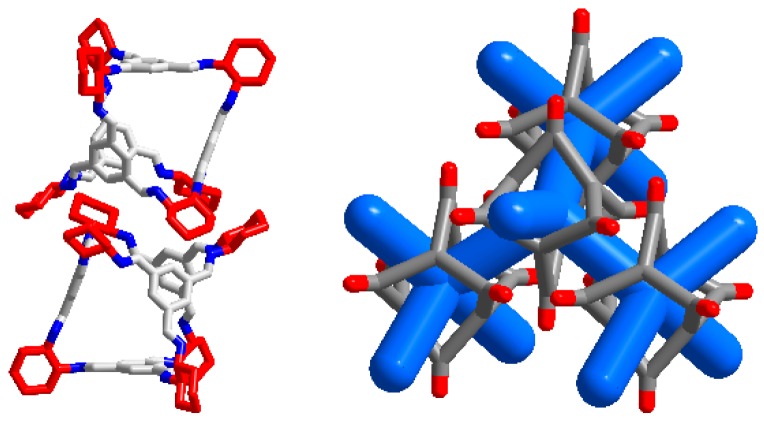
Schematic diagram of the structure of CC3-R.

As versatile functional material platforms, porous organic cages (POCs) have attracted much attention [10,11,12,13], and have been widely applied in various areas such as gas-selective adsorption and separation [14,15,16,17], molecular recognition [18,19,20,21,22,23,24,25,26,27], catalysis [28], water treatment [29] and sensing [30]. As shown in Figure 1, the *R*-type chiral POC CC3-R has an interlinked chiral pore channel structure with adjacent tetrahedral cages packed together [31,32]. The chiral pore channel structures and cyclohexyl, imino and phenyl groups of cage molecules constitute a chiral microenvironment comprising a variety of enantioselective factors including dispersion forces, dipolar interactions and hydrogen bonds [33]. These properties combine to make CC3-R an excellent chiral selector for use in chiral recognition methods. Recently, a CC3-R-modified GC stationary phase was developed for the separation of racemates, and exhibited excellent enantioselectivity [34].

**Figure 2:**
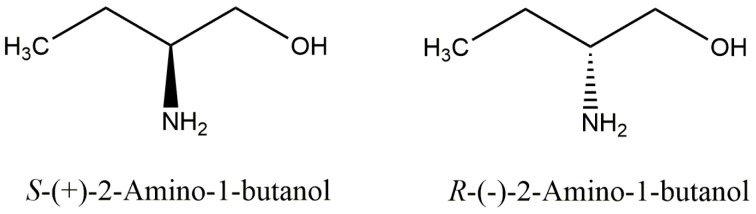
Molecular structure of 2-amino-1-butanol.

2-Amino-1-butanol (Figure 2) is generally used as an intermediate in the synthesis of pharmaceuticals such as the bacteriostatic antituberculosis agent (*S*,*S*)-ethambutol [35,36,37,38]. In the present work, CC3-R was applied as a chiral selector in PVC membrane electrodes, resulting in impressive enantioselectivity for *S*-2-amino-1-butanol. Factors influencing the enantioselectivity of the CC3-R-based membrane electrode, such as the content of CC3-R, the category of plasticiser and the pH value of analyte solutions, were systematically investigated.

## 2. Results and Discussion

### 2.1. Characterisation of the Synthesised CC3-R

**Figure 3:**
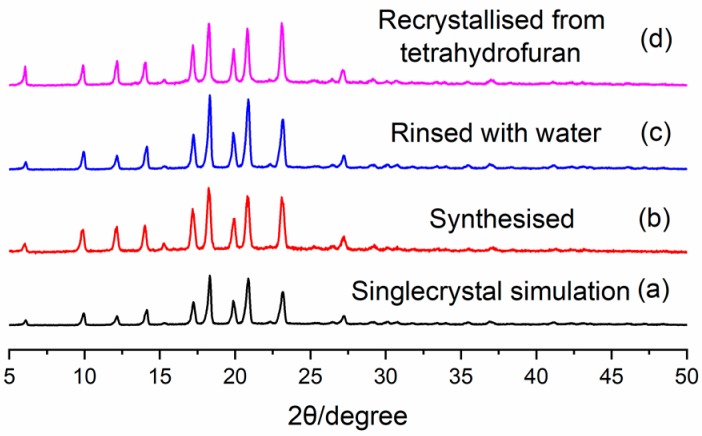
PXRD patterns of CC3-R: (**a**) Singlecrystal simulation, (**b**) synthesised, (**c**) rinsed with water for 48 h, (**d**) recrystallised from tetrahydrofuran.

The synthesised CC3-R crystals were characterised by Nuclear Magnetic Resonance (NMR), Powder X-ray diffraction (PXRD) and elemental analysis. As can be seen in Figure 3, the PXRD pattern of synthesised CC3-R crystals was consistent with the Singlecrystal simulation. Furthermore, CC3-R retained the same crystallinity and structure whether recrystallised from tetrahydrofuran or rinsed with water for 48 h, demonstrating excellent stability as chiral selector in the membrane electrode.

Elemental analysis was performed on CC3-R (C_72_H_85_N_12_); calculated = C 77.31, H 7.66, N 15.03; detected = C 77.08, H 7.76, N 14.88. ^1^H-NMR (CDCl_3_) δ = 8.18 (s, 12H, -C*H*=N-), 7.92 (s, 12H, -Ar-*H*), 3.36 (m, 12H, -CHN-), 1.86–1.54 (m, 48H, -CH_2_-) ppm. ^13^C-NMR (CDCl_3_) δ = 159.15, 136.64, 129.53, 74.65, 33.02, 24.39 ppm. All characterisation data confirmed that CC3-R was successfully synthesised.

### 2.2. Optimisation of Membrane Components

The nature and amount of chiral selector and plasticiser contained in the membrane can strongly influence the selectivity and sensitivity of the membrane electrode. Consequently, the potential response characteristics of multiple electrodes with different quantities of CC3-R and three types of plasticiser (*o*-NPOE, DOS and DBP) were evaluated.

**Figure 4:**
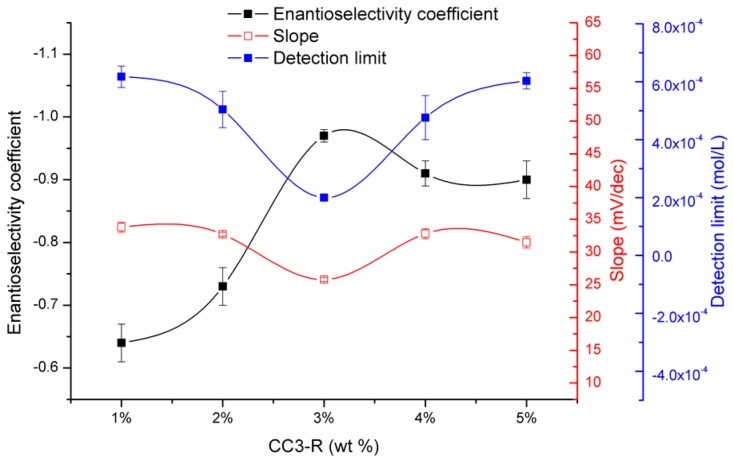
Potential response characteristics of membrane electrodes with different CC3-R contents.

Figure 4 shows the potential response characteristics of membrane electrodes with different CC3-R mass percentages. The performance of the membrane electrode improved with increasing CC3-R content, and the best enantioselectivity toward *S*-2-amino-1-butanol was achieved with 3% CC3-R (by weight). However, the enantioselectivity and sensitivity decreased slightly when the amount of CC3-R reached 4%. It is possible that the PVC membrane becomes saturated, hence the number of recognition sites does not increase proportionately with the chiral selector. Moreover, excess CC3-R could affect the ion-exchange capacity of the membrane electrode.

**Figure 5:**
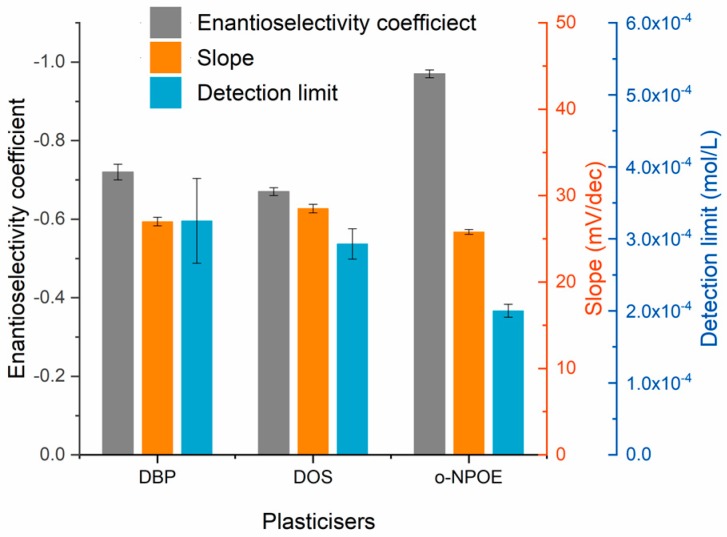
Potential response characteristics of membrane electrodes with different types of plasticiser.

The influence of the type of plasticiser is shown in Figure 5. DOS and DBP were clearly inferior to *o*-NPOE in terms of detection limit and enantioselectivity coefficient for *S*-2-amino-1-butanol.

### 2.3. Effect of pH on the Electrode

**Figure 6:**
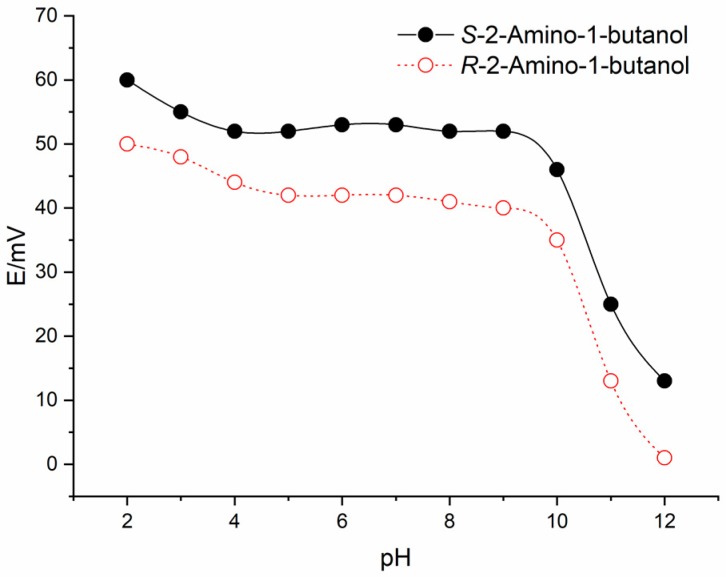
The influence of pH on the response performance of the membrane electrode to *S*/*R*-2-amino-1-butanol.

In order to investigate the effect of pH on the response performance of the optimised membrane electrode, the potential response value of the 2-amino-1-butanol solution (1.0 × 10^−3^ mol·L^−1^) was measured at different pH values (pH 2.0–12.0). As shown in Figure 6, the potential response value was stable within a pH range of 5.0–9.0. Furthermore, a large difference between the two enantiomers was observed at pH 9.0. Therefore, pH 9.0 was adopted for measurements using the optimised membrane electrode.

### 2.4. Enantioselectivity Coefficient of the Electrode

Figure 4 shows $\log K_{S,R}^{Pot}$ values for membrane electrodes of varying composition. The optimised membrane electrode containing 3 wt% CC3-R displayed impressive enantioselectivity toward *S*-2-amino-1-butanol over *R*-2-amino-1-butanol ($\log K_{S,R}^{Pot}$ = −0.98). By comparison, the CC3-S (3 wt%) modified membrane electrode showed similar enantioselectivity toward *R*-2-amino-1-butanol ($\log K_{S,R}^{Pot}$ = −0.94).

Furthermore, the $\log K_{S,\mathrm{int}}^{Pot}$ value was used to evaluate the enantioselectivity of the optimised electrode in the presence of interfering ions with a similar configuration to 2-amino-1-butanol [39]. Specifically, the potential values of *R*/*S*-2-amino-3-phenyl-1-propanol, *R*/*S*-2-amino-3-methyl-1-butanol and *R*/*S*-3-amino-1,2-propanediol (0.1 mol·L^−1^) were measured, and $\log K_{S,\mathrm{int}}^{Pot}$ values are shown in Table 1.

As shown in Table 1, the membrane electrode exhibited comparable responses to other alkamines with similar configurations to 2-amino-1-butanol. Steric hindrance caused by additional organic groups of other alkamines could impair the recognition performance during ion exchange. The electrode displayed slight enantioselective recognition of enantiomers of 2-amino-3-methyl-1-butanol, which have the most similar configuration. However, 2-amino-3-methyl-1-butanol yielded similar potential response values, and caused significant interference.

### 2.5. Recognition of Mixing Samples

**Figure 7:**
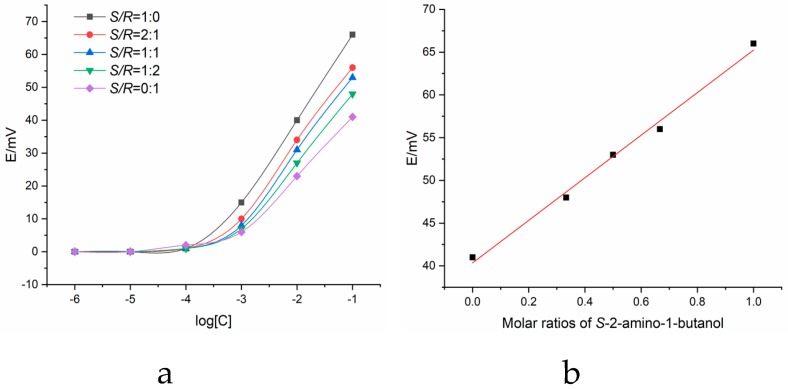
(**a**) Potential responses of *S*- and *R*-enantiomers of 2-amino-1-butanol mixing solutions. (**b**) Positive linear correlation between the proportion of *S*-2-amino-1-butanol and potential response values of mixing solutions (0.1 mol·L^−1^).

To further explore the enantioselectivity of the developed membrane electrode, a mixing sample test was conducted using different molar ratios of *S*- and *R*-enantiomers of 2-amino-1-butanol (Figure 7). The results showed that the potential response values of the mixing solution increased with increasing proportion of *S*-2-amino-1-butanol, revealing a clear positive linear correlation between the proportion of *S*-2-amino-1-butanol and potential response values of mixing solutions (0.1 mol·L^−1^). These results demonstrate the selective recognition of *S*-2-amino-1-butanol in the presence of *R*-2-amino-1-butanol.

## 3. Materials and Methods

### 3.1. Materials

Enantiomers of 2-amino-1-butanol, 2-amino-3-phenyl-1-propanol, 2-amino-3-methyl-1-butanol, and 3-amino-1,2-propanediol were obtained from Aladdin (Shanghai, China). (*R*,*R*)-1,2-Diaminocyclohexane and 1,3,5-triformylbenzene were purchased from Acros (Geel, Belgium). *o*-Nitrophenyl Octyl Ether (*o*-NPOE), dioctyl sebacate (DOS) and dibutyl phthalate (DBP) were obtained from TCI (Tokyo, Japan). Polyvinyl chloride (PVC) powder and trifluoroacetic acid were purchased from Sigma-Aldrich (St. Louis, MO, USA). All other reagents were of analytical grade. Deionised water was used to prepare and dilute all buffer and analyte solutions.

### 3.2. Synthesis of CC3-R

CC3-R was synthesised using a previously reported method [29]. Briefly, 20 mL dichloromethane was added dropwise onto 1.0 g 1,3,5-triformylbenzene in a two-necked flask without stirring at room temperature, and 20 μL trifluoroacetic acid was added as a catalyst. Within minutes, 20 mL dichloromethane containing 1.0 g (*R*,*R*)-1,2-diaminocyclohexane was dripped slowly into the mixture. After reaction for 72 h at room temperature, white crystals were present on the wall of the flask, which were filtered and rinsed with ethanol/dichloromethane (95:5 *v*/*v*).

### 3.3. Preparation of Enantioselective Membrane Electrodes

**Scheme 1:**
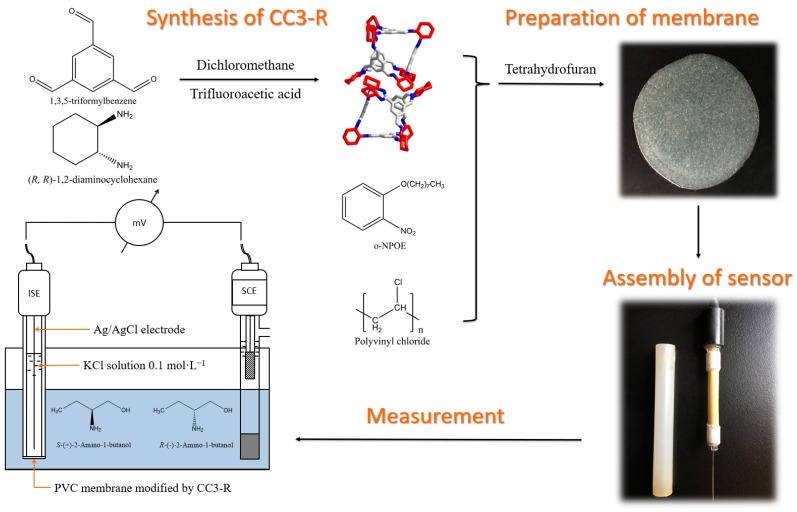
Schematic illustration of enantioselective potentiometric sensor fabrication.

To prepare the PVC membranes, PVC powder, plasticiser (*o*-NPOE), and CC3-R were added to 3 mL tetrahydrofuran and stirred to form a transparent solution [40]. This was poured onto a glass sheet and volatilised for 24 h to form a semitransparent film ~0.5 mm thick. The obtained film was incised into an appropriately sized disc and assembled using a PVC tube, which was subsequently filled with 0.1 mol·L^−1^ KCl as an internal reference solution. A silver chloride electrode was applied as an internal reference electrode, and a saturated calomel electrode was utilised as a reference electrode. For comparison, a CC3-*S*-modified membrane electrode was prepared in the same way. The overall strategy for enantioselective potentiometric sensor fabrication is depicted in Scheme 1.

### 3.4. Potentiometric Measurement

The direct potentiometric method was applied to measure the potential value of each *S*/*R*-2-amino-1-butanol solution at different molar concentrations (1.0 × 10^−6^ to 1.0 × 10^−1^ mol·L^−1^) and mixing solution with different molar ratios (*S*/*R* = 1:0, 2:1, 1:1, 1:2, and 0:1). A Model PHS-3C pH meter (Leici, Shanghai, China) was used for potentiometric and pH measurements, and all potentiometric measurements were performed during stirring at room temperature. Before measurement, membrane electrodes were soaked in *S*-2-amino-1-butanol solution (1.0 × 10^−3^ mol·L^−1^) for 24 h.

A revised separate solution method was used to calculate the enantioselectivity coefficient ($\log K_{S,R}^{Pot}$) with the following formula:

$$\log K_{S,R}^{Pot}=\frac{E_{R}-E_{S}}{D}$$

where *E_R_* and *E_S_* are the potentials of 0.1 mol·L^−1^ *R*- and *S*-2-amino-1-butanol solutions, respectively, and *D* is the slope of the response curve of *S*-2-amino-1-butanol.

## 4. Conclusions

The chiral porous organic cage CC3-R proved to be a useful chiral selector for the modification of PVC membrane electrodes to generate enantioselective potentiometric sensors. The optimised membrane electrode containing 3 wt% CC3-R exhibited enantiomeric recognition toward *S*-2-amino-1-butanol over *R*-2-amino-1-butanol ($\log K_{S,R}^{Pot}$ = −0.98) with acceptable sensitivity, and a near-Nernst response of 25.8 ± 0.3 mV/decade toward *S*-2-amino-1-butanol at pH 9.0.

## Figures and Tables

**Table 1:** $\mathrm{Log}K_{S,\mathrm{int}}^{Pot}$ values of ions potentially interfering with *S*-2-amino-1-butanol.

Interference Ion	$\mathrm{Log}K_{S,\mathrm{int}}^{Pot}$
*R*-2-Amino-1-butanol	−0.98
*S*-2-Amino-3-phenyl-1-propanol	−0.59
*R*-2-Amino-3-phenyl-1-propanol	−0.59
*S*-2-Amino-3-methyl-1-butanol	0.31
*R*-2-Amino-3-methyl-1-butanol	0.26
*S*-3-Amino-1,2-propanediol	−0.41
*R*-3-Amino-1,2-propanediol	−0.41
